# Interpretation of gene associations with risk of acute respiratory distress syndrome: *P* values, Bayes factors, positive predictive values, and need for replication

**DOI:** 10.1186/s13054-016-1550-8

**Published:** 2016-12-21

**Authors:** Sebastian Rimpau, Ari R. Joffe

**Affiliations:** Department of Pediatrics, Division of Pediatric Critical Care; and Stollery Children’s Hospital, University of Alberta, 5-456 Edmonton Clinic Health Academy, 11405 87 Avenue, Edmonton, Alberta T6G 1C9 Canada

**Keywords:** Acute respiratory distress syndrome, Bayes factor, Gene association, *P* values

Single nucleotide polymorphisms (SNPs) in certain genes play a role in the observed variability in development and severity of acute respiratory distress syndrome (ARDS). Identified SNPs can direct future studies aiming to target diagnostic, preventive, and therapeutic interventions for the complex pathophysiology of ARDS [1]. For example, CFTR is involved in fluid absorption from alveoli and in negatively modulating the inflammatory response [2, 3], and Perez-Marques et al. report that SNPs in DNA for proteins involved in splicing in the exon 9 region of CFTR mRNA were independently associated with risk for ARDS [2]. The same group have identified other statistically significant candidate gene associations with the risk for pediatric ARDS (Table 1) [2–7]. In adults, other candidate gene associations with risk for development of and outcome from ARDS have been suggested [1, 8]. How should these gene-association hypotheses be interpreted?

**Table 1:** Studies to identify risk, understand pathophysiology, and provide insight into newer individualized therapies for severity of CAP-induced ARDS in children

Study (year)	Patient group (n)	Time frame (years)	Gene variants examined	OR (unadjusted)	OR (adjusted)	Comment
[4] (2008)	African American (515) and Caucasian (232)	March 2004 to August 2006; 2 centers	IL-1ra: absent A1 allele	MV 8.6% vs no-MV 2.6% (*P* = 0.003)	OR 2.65, *P* = 0.046	To predict MV (*n* = 96). Not statistically significant if African Americans and Caucasians analyzed separately
				ARDS 9.1% vs no-ARDS 2.9%, *P* = 0.023	OR 3.10, *P* = 0.052	To predict ARDS (*n* = 49)
[5] (2010)	African American (443)	Not stated	4 MYLK SNPs	*P* = 0.39 to 1.0	-	To predict MV (*n* = 41) and ARDS (*n* = 28)
	Caucasian (253)	Not stated	5 MYLK SNPs	*P* = 0.26 to 1.0	-	To predict MV (*n* = 33) and ARDS (*n* = 19)
[6] (2011)	African American (395)	March 2004 to August 2006; 2 centers	7 SP-B SNPs rs1130866 rs3024793	*P* = 0.016 *P* = 0.030	OR 2.27, *P* = 0.040 OR 3.00, *P* = 0.012	To predict MV (*n* = 37) Not statistically significant if adjust for bacterial culture positive
			rs7316	*P* = 0.06	OR 2.95, *P* = 0.031	To predict ARDS (*n* = 26)
[3] (2012)	African American (474)	Not stated; 3 centers	CFTR 2 low risk vs 1 or 2 high risk [(TG)_≥12_T_≤5_] alleles	*P* = 0.0013	OR 3.19, *P* = 0.0007	To predict MV (*n* = 43)
				*P* = 0.0061	OR 3.36, *P* = 0.0032	To predict ARDS (*n* = 29). Nasal swab was done in 113 and positive for a virus in 43 (38%)
	Caucasian (304)	Not stated; 3 centers	As above	*P* = 0.21	Not statistically significant	To predict MV (*n* = 42)
				*P* = 0.84	Not statistically significant	To predict ARDS (*n* = 32). Nasal swab was done in 89 and positive for a virus in 22 (25%)
[7] (2014)	African American (443)	Not stated; 3 centers	Caspase-12 long allele	*P* = 0.83 and 0.48	-	For MV (*n* = 41), ARDS (*n* = 28). Also reported for: mortality (*n* = 5), severe sepsis (*n* = 17), vasopressor use (*n* = 15), renal dysfunction (*n* = 11), hematologic dysfunction (*n* = 9)
[2] (2016)	African American (474)	Not stated; 3 centers	Splicing factors of CFTR mRNA (7 genes, 66 SNPs)	19 SNPs in 6 genes with *P* < 0.20	3 SNPs in CELF-2 (OR 2.95, 4.28, and 2.66 with *P* = 0.032, 0.004, and 0.044). 2 SNPs in TIA-1 (OR 3.70, 5.42 with *P* = 0.005, 0.018)	ARDS (*n* = 29). Also (TG)_≥12_T_≤5_ allele (OR 3.01, *P* = 0.012) and age ≥11 years (OR 14.9, *P* < 0.0001)
	Caucasian (304)	Not stated; 3 centers	7 genes, 41 SNPs	8 SNPs in 4 genes with *P* < 0.20	1 (different) SNP in CELF-2 (OR 3.22, *P* = 0.014). No TIA-1 SNPs or (TG)_≥12_T_≤5_ alleles	ARDS (*n* = 32). Also age ≥11 years (OR 9.20, *P* < 0.0001) and asthma (OR 0.20, *P* = 0.04)

*ARDS* acute respiratory distress syndrome (including PaO_2_/FIO_2_ ≤ 300), *CELF-2* elav-like family member 2, *CFTR* cystic fibrosis transmembrane conductance regulator, *IL1-ra* interleukin 1 receptor antagonist, *MV* invasive (endotracheal tube or tracheostomy) or noninvasive (nasal prongs or face mask)—oxygen delivered by low or high flow via nasal cannula was not considered MV [4], *MYLK* myosin light chain kinase, *OR* odds ratio, *SNP* single nucleotide polymorphism, *SP-B* surfactant protein B, *TIA-1* T-cell intracellular antigen 1

Quality control: 5–10% genotyped a second time; blinded analysis of genotype to clinical status (but also state; two individuals independently assessed the results from the analyses and assigned genotypes [4]). Comparisons are between 0 and 1 versus 2 copies; or sometimes 0 versus 1 or 2 copies

## Theoretical considerations: when is a gene-association hypothesis supported?

The *P* value is the probability, assuming that the null hypothesis (i.e., no difference between groups) is in fact true and that all model assumptions (i.e., no selection, attrition, analysis, or reporting bias—only chance is operating) are satisfied, of obtaining a result equal to or more extreme than what was actually observed [9, 10]. The “*P* value fallacy” is “the illusion that conclusions can be produced with certain ‘error rates’ without consideration of information from outside the experiment” [9]. The fallacy is to think that the *P* value refers to a *hypothesis* probability, involving inductive reasoning back from evidence (observations) to underlying truth [9–11]. This leads to misinterpretations of the *P* value (Table 2). To make the inductive inference about hypothesis probability requires Bayesian methods.

**Table 2:** Some surprising statements about *P* values, results of Bayesian methods, and empirical evidence supporting the predictions of Bayesian methods

Surprising *P*-value misinterpretations (false statements)	Correction	Reference
The *P* value is the probability that the null hypothesis is true	The *P* value assumes the null hypothesis is true	[10]
* P* ≤ 0.05 means the null hypothesis is false, or should be rejected	* P* ≤ 0.05 simply flags the data as being unusual if all the assumptions used to compute it were correct	[10]
* P* > 0.05 means the null hypothesis is true, or should be accepted	*P* > 0.05 only suggests that the data are not unusual if all the assumptions used to compute it were correct; the same data would also not be unusual under many other hypotheses	[10]
If you reject the null hypothesis because *P* ≤ 0.05, the chance your “significant finding” is a false positive is 5%	The *P* value only refers to how often you would be in error over very many uses of the test across different studies, and not in a single use of the test	[10]
Surprising results of Bayesian methods		
In late-phase clinical trials with equipoise (the prior probability of the null hypothesis is 50%), a study with a *P* = 0.05 makes the posterior probability of the null hypothesis no less than 13%		[11]
In more exploratory research (the prior probability of the null hypothesis is, say, 75%), a study with a *P* = 0.05 or *P* = 0.01 makes the posterior probability of the null hypothesis no less than 31% and 10%, respectively		[11]
An adequately powered (80%) exploratory epidemiologic (prior 1:10, bias 0.3, α = 0.05) study with a statistically significant finding has a positive predictive value (PPV) 20% and, if underpowered (20%), a PPV of 10%		[12]
In large traditional cohort studies (prior 1:20, bias 0.1, α = 0.05, power 90%), the false positive to false negative ratio of findings is 32:1		[13]
In a well done (power 95%, α = 0.05) cohort study testing SNPs with less than compelling evidence (prior 1:100), with a statistically significant finding (*P* = 0.05 or 0.01) the PPV is 16.1% and <60%; even with fairly compelling prior evidence (prior 1:10), the PPV is 67.9% and <90%		[14]
Surprising empirical evidence supporting the predictions of Bayesian methods		
In traditional genome epidemiology [a “few candidate risk factors are selected based on diverse considerations” (low prior); small sample size (low power, given the small size of expected effect); “discovery hunting using conventional levels” of statistical significance, confounding, selective reporting (bias)], the crude replication rate of statistically significant genetic associations is ~1.2%		[13]
Hallmarks of discovery exploratory research (low priors, low BF, high bias): “vibration of effects” (evidence of inflated early effect sizes in epidemiologic associations), “Proteus phenomenon” (a rapid early sequence of extreme, opposite results in retrospective hypothesis-generating molecular research), and “winners curse” (the first positive study provides inflated estimates compared to reality)		[12, 13]

Bayesian methods are *conceptually* simple: (Prior-odds of null hypothesis)(Bayes factor) = (Posterior-odds of null hypothesis) [9]. The prior-odds are based on evidence external to the study concerning the plausibility of the null hypothesis; in a field of study, this is the ratio of the number of “true relationships” to “no relationships” among those tested in the field [12]. The Bayes factor (BF) measures the relative support, from the observed evidence, for two hypotheses: (Probability of the data given the null hypothesis)/(Probability of the data given the alternative hypothesis). The BF modifies the prior probability to give the posterior probability of the null hypothesis (or, if one reverses the numerator and denominator, the post-study probability that there is a true association: positive predictive value (PPV)). One can calculate, from the same numbers used to calculate the *P* value, the minimum BF: the strongest evidence against the null hypothesis, using the best supported hypothesis (the observed association) as the alternative-hypothesis [11]. One can also calculate the PPV of a statistically significant finding using the prior probability of an association, the BF based on power and alpha [BF = αβ/(1 − α)(1 − β)], in addition to bias (affecting the accuracy of the alpha and also reflecting our estimate of the prior-odds) [12–14]. The PPV is lowered by low study power (smaller studies with small expected effect sizes), low pre-study odds (hypothesis-generating experiments), bias (flexibility in designs, definitions, outcomes, and analytic modes), and number of teams working in the field (hotter scientific fields) [12]. There are some surprising results of Bayesian methods (Table 2).

### Empirical considerations: when is a gene-association hypothesis supported?

There is evidence to support the predictions from Bayesian methods in interpreting study results (Table 2). This is particularly so in genetic-association studies where the expected true (when there is a true association) odds ratios for common SNPs with common complex diseases (such as ARDS) is repeatedly found to be 1.1–1.4; this means that studies have low power unless there are >1000 subjects [12, 15]. This empirical evidence (Table 2) suggests that Bayesian methods, which keep statistical evidence (conveyed traditionally by the *P* value and more usefully by the BF) distinct from inductive inferences about hypotheses, are useful because they incorporate data external to the study (estimation of priors) in order to arrive at a conclusion about a hypothesis (posterior probability of the probed association being true) [9–12].

### Interpreting ARDS gene-association studies

Using the growing cohort of patients, six ARDS gene-association studies have been published by this group (Table 1) [2–7]. These reports were well done according to reporting guidelines [15]. We ask three questions to improve interpretation of these (and, in general, future) gene-association studies in critical care.

1. Priors: how likely is an association to be expected given information external to the study? Considerations are listed in Table 3. In gene-association studies for complex diseases, the prior is usually in the range of 0.001 (SNPs with only limited prior evidence) to 0.1 (SNPs that already show fairly compelling evidence for association), and in non-replication studies is likely closer to 0.001 [14].

**Table 3:** Considerations relevant to interpretation of the results of gene-association studies for the risk of ARDS

Question	Considerations	References
What is the prior probability of the gene association?	Pathophysiology of ARDS is very complex	[1]
	Severity of ARDS likely also depends on the inciting cause (e.g., pathogen) and its duration prior to appropriate treatment	[15]
	Many gene-association studies are the first to examine for an association between the particular genes with the development of ARDS in a hot field of interest	[12, 13]
	It may be unknown if the SNPs are associated with changes in their respective protein levels or function (e.g., there is no change in the amino acid sequence of the CELF-2 protein)	[2]
	The rationale for exploration for a gene association is often based on limited prior information (e.g., the current study was done because of the previous finding, in one study with the same cohort, that CFTR gene variants are associated with ARDS in African American children with CAP)	[3, 13, 14]
What is the minimum BF observed, or the PPV?	The minimum BF for a *P* = 0.03 and *P* = 0.01 are 0.095 and 0.036, respectively. This modifies the prior: even if very high (e.g., prior of 0.25 for the alternative hypothesis), the null hypothesis probability is lowered to no less than 22% or 10%, respectively	[11]
	The PPV, assuming a prior of 0.01, power 0.80, and *P* values between 0.001 to 0.01, is between 55–90%; assuming a more realistic prior of 0.001, the PPV is 10–50%	[14]
	Given the expected odds ratios of gene association for complex diseases such as ARDS are <1.5, and the often low number of patients with ARDS, the power of the study was well below 0.5. This lowers the PPV even more	[12, 15]
How much bias occurred?	Attrition bias: not all the cohort has genotyping done. In this example: -The number of patients is often lower in more recent times	[15]
	Selection bias: flexibility in eligibility criteria due to different definitions of conditions. In this example: -CAP definition required at least two of tachypnea, dyspnea, or hypoxemia, but in one study the list also included cough or abnormal chest exam (selection bias)	[6, 12, 13, 15]
	Analysis and reporting biases: flexibility in definitions of predictor variables, in decisions of which covariates to adjust for, in decisions of outcomes to examine, and in which analyses to report. In this example:	[12, 13, 15]
	a. Predictor variables definitions: only in the CFTR studies was age categorized as <11 versus ≥11 years; the cohorts were analyzed separately, although in other studies African Americans and Caucasians were combined or only results for African Americans were reported	[2, 3, 4, 6, 7]
	b. Covariates to adjust for: in the one study where it was examined, a statistically significant association was “lost” if adjusted for bacterial culture positivity; asthma was forced into the multiple regressions in only this most recent study; other known SNPs were not adjusted for (especially those the same group previously found associated with ARDS: IL-1ra A1 allele, SP-B); duration of symptoms prior to enrolment, treatment (e.g., antibiotics), and duration of treatment are not reported or adjusted for	[2, 4, 6]
	c. Outcomes to determine and report: the outcome was ARDS, but in other studies also included mechanical ventilation, severe sepsis, vasopressor use, and renal or hematologic dysfunction, and could include hospital admission, PICU admission, and duration of hospitalization	[3, 4–7]

*ARDS* acute respiratory distress syndrome, *BF* Bayes factor, *CAP* community acquired pneumonia, *CELF-2* elav-like family member 2, *CFTR* cystic fibrosis transmembrane conductance regulator, *IL1-ra* interleukin 1 receptor antagonist, *PPV* positive predictive value, *SNP* single nucleotide polymorphism, *SP-B* surfactant protein B
2. Minimum BF and PPV: what does the evidence from the study show us? Considerations are listed in Table 3. Assuming a *P* value threshold 0.01 to 0.001, for a prior of 0.01 the PPV is (assuming power of 0.5) 38–82%, and for a prior of 0.001, 8–40% [14].
3. Bias: do we need to modify these estimates for study bias? Considerations are listed in Table 3. If bias is low (0.05–0.2 = “the proportion of probed analyses that would not have been ‘research findings’, but nevertheless end up presented and reported as such, because of bias”), power is 50%, and prior is very high (0.1), the PPV that a statistically significant finding is true is 35–55% [12].

## Conclusions

The observed evidence (the *P* value, or better yet, the BF) can be combined with prior considerations of plausibility to determine how well two hypotheses are supported (posterior probability, PPV). The posterior probability (PPV) that there is an association between exploratory SNPs and severity of ARDS in children is low given the low prior probability, the modest BF (reflected in modest *P* values and power), and potential for bias. This is not necessarily a problem if our interest is in generating hypotheses for further scientific study [13]. An interesting hypothesis has been suggested (i.e., a gene association) and warrants further investigation; we should wait for replication in additional larger studies before accepting this hypothesis. These future studies will have a prior probability that is closer to 0.1 (the posterior probability after the current study), and thus replication would move us much further toward accepting the hypothesis [13–15]. Overall, caution is warranted: most genetic associations for ARDS in adults have not replicated [8].

## Abbreviations

ARDS: Acute respiratory distress syndrome
BF: Bayes factor
CAP: Community-acquired pneumonia
CELF2: Elav-like family member 2
CFTR: Cystic fibrosis transmembrane conductance regulator
PPV: Positive predictive value
SNP: Single nucleotide polymorphism
TIA-1: T-cell intracellular antigen-1
